# EHMTI-0023. Characteristics of post-traumatic headache following mild traumatic brain injury in military personnel in Iran

**DOI:** 10.1186/1129-2377-15-S1-D49

**Published:** 2014-09-18

**Authors:** S Rezaei Jouzdani, G Kaka

**Affiliations:** 1 Neuroscience Research Center, Baqiyatallah University of Medical Sciences, Isfahan, Iran; 2 Neuroscience Research Center, Baqiyatallah University of Medical Sciences, Tehran, Iran

## Objectives

The primary goal of this study was to evaluate the incidence and characteristics of post-traumatic headache(PTH) attributed to mild brain injury in military personnel in Iran within a prospective and observational study design.

## Methods

A prospective observational study was conducted with a cohort of military personnel under military education during 6 months period at Amiralmomenin Military education center in Isfahan in Iran. Through all military personnel under education 322 personnel were selected randomly in simple manner were given 13-item Mild brain injury questionnaire accompany with affective disorders and headache questionnaires and were reevaluated in 3 months interval.

## Results

A total of 30 (9.3%) of 322 military personnel met criteria for a mild brain injury. Among them 18 personnel (60%) reported having headaches during 3-month of reevaluation. Patients with affective disorders such as post-traumatic stress disorder (PTSD) and depression were at higher risk for developing PTH following mild brain injury (p<0.05). PTH did not relate to demographic factors such as age or type of trauma. At follow up, PTH had abated in 3 months except in 2 patients developed chronic PTH pattern. We did not observe any significant difference of medications prescribed by practitioners between different classified patterns of PTH (p>0.05).

## Conclusions

PTH attributed to mild brain injury is a common disorder in military personnel. Migrainous features are predominant among them in comparison with general population. PTH is not related to type of trauma but has association with affective disorders.

No conflict of interest.

